# Himalayan Mushrooms as a Natural Source of Ergosterol and Vitamin D_2_: A Review of Nutraceutical and Functional Food Perspectives

**DOI:** 10.3390/foods14203516

**Published:** 2025-10-15

**Authors:** Pooja Panthari, Garima Khantwal, Manoj Kumar, Xiaomin Shang, Ji-Ho Lee, Soha Haniyyah, Kavita Sharma, Ramesh Kumar Saini

**Affiliations:** 1 School of Health Sciences and Technology, UPES, Dehradun 248007, Uttarakhand, India; pooja.128461@stu.upes.ac.in (P.P.); garima.130264@stu.upes.ac.in (G.K.); 2 Forest Pathology Discipline, Forest Protection Division, ICFRE—Forest Research Institute, Dehradun 248007, Uttarakhand, India; manojk@icfre.org; 3 Jilin Provincial Key Laboratory of Nutrition and Functional Food, Jilin University, Changchun 130062, China; xmshang@jlu.edu.cn; 4 Department of Biological Environment, School of Natural Resources and Environmental Science, Kangwon National University, Chuncheon 24341, Republic of Korea; micai@kangwon.ac.kr; 5 Department of Biological Sciences, Idaho State University, Pocatello, ID 83209, USA; sohahaniyyah@isu.edu; 6 Department of Biomedical and Pharmaceutical Sciences, Idaho State University, Pocatello, ID 83209, USA

**Keywords:** ergosterol, UV-irradiation, ergocalciferol, 25-hydroxyvitamin D_2_, *Morchella* sp., *Agaricus* sp.

## Abstract

Mushroom diversity is essential for maintaining ecological balance and provides valuable bioactive compounds for human use. Beyond their nutritional value, mushrooms contribute to functional foods and have applications in nutraceuticals, pharmaceuticals, and biotechnology. For example, β-glucans from *Lentinula edodes* are commercialized as immune-enhancing nutraceuticals, polysaccharide Krestin (PSK) from *Trametes versicolor* is used as an adjuvant in cancer therapy, and enzymes such as laccases from *Pleurotus* species are widely applied in biotechnological processes. One of the abundant compounds found in mushrooms is ergosterol, which is a sterol present in the cell membrane of the fungal body. Ergosterol has significant health benefits due to its antioxidant, immunomodulatory, and anti-inflammatory properties. Furthermore, ergosterol is a precursor to vitamin D_2_ (ergocalciferol), which can be synthesized through exposure to ultraviolet (UV) light and thermal radiation. This review highlights the importance of Himalayan mushroom biodiversity, particularly the wild edible mushrooms traditionally collected and used. This review thoroughly discusses the ergosterol and vitamin D_2_ content, their biosynthesis in mushrooms, and the role of environmental factors used to enhance biosynthesis. We also discuss the sustainable cultivation of Himalayan mushrooms and their nutraceutical properties. Several Himalayan mushrooms have been reported to possess health-promoting properties, and their incorporation into functional foods may contribute to improved public health. Furthermore, the future research directions are highlighted.

## 1. Introduction

The Himalayas are a world biodiversity hotspot due to their diverse flora and fauna from various climatic conditions and altitudes [1,2]. The unique biodiversity is derived from the extreme climate; therefore, some species are still unknown. Among all such biodiversities, mushrooms hold great nutritional, medicinal, and ecological importance [3,4,5,6]. Mushrooms, also known as macro-fungi, are the fruiting bodies of fungi, primarily basidiomycetes and some ascomycetes. Basidiomycetes comprise approximately 30,000 described species worldwide, including members of *Agaricus*, puffballs, bracket fungi, chanterelles, as well as plant-parasitic rusts and smuts. In contrast, *Ascomycota* comprises at least 40,000 species, some of which are not readily noticeable, as well as familiar groups such as morels, truffles, cup fungi, certain lichens, and numerous microscopic molds and yeasts. Macrofungi are estimated to include up to 140,000 species globally, of which only 16–41% have been scientifically documented [7,8]. Within this known fraction, 2189 are edible, with 2006 considered safe for direct consumption and 183 requiring pretreatment to remove toxic components [9]. The large world of unknown mushroom species represents a scientific challenge. It highlights the untapped potential of mushrooms in various areas, particularly in nutrition and medicine [10,11].

The unique environmental attributes of the mountains contribute to the wild mushrooms that locals have traditionally used for medicinal and dietary purposes [12,13]. These mushrooms are rich in bioactive compounds with demonstrated nutritional and therapeutic properties. Documented activities include antioxidant, antimicrobial, antidiabetic, anticancer, and immunomodulatory effects, although such properties are species-specific rather than universal across all mushrooms [14,15,16,17,18,19,20,21]. Major classes of bioactive metabolites identified in mushrooms include polyphenols, polysaccharides, sterols, terpenoids, and β-glucans [22,23,24].

One of the most abundant sterols in mushrooms is ergosterol, which remains insufficiently investigated, particularly in wild species from the Himalayas. Ergosterol is the principal sterol of fungal and yeast membranes, where it contributes to biophysical stability by regulating lipid packing, modulating bilayer fluidity, and maintaining structural integrity under stress conditions [25]. Apart from its structural role, ergosterol is also known for its bioactivity, antifungal, antioxidant, immunomodulatory, and anti-inflammatory properties, making it a medicinal and nutraceutical compound [26,27,28]. In addition to ergosterol, mushrooms contain several minor sterols [29]. Notably, the specific profiles of these minor sterols when analyzed through chromatographic and spectroscopic techniques (e.g., gas chromatography-mass spectrometry) can serve as reliable markers for mushroom species authentication [29,30,31].

**Figure 1:**
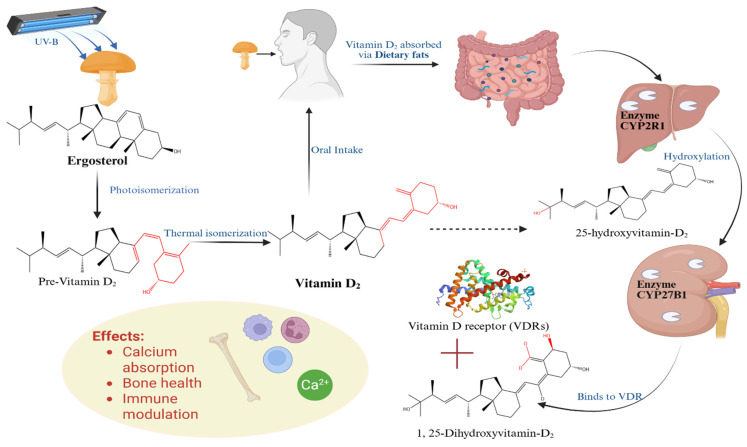
The schematic representation of UV-B-induced photoisomerization of ergosterol to pre-vitamin D_2_, thermal conversion to vitamin D_2_, and subsequent metabolic activation in humans via hepatic (CYP2R1) and renal (CYP27B1) hydroxylation, leading to binding of the active metabolite [1,25(OH)_2_D_2_] to the vitamin D receptor (VDR).

Furthermore, ergosterol is a precursor to a critical vitamin D_2_ (ergocalciferol), which can be synthesized through exposure to ultraviolet (UV) light and thermal radiation [32,33]. Under controlled UV-B treatment, ergosterol in mushrooms undergoes photoisomerization to pre-vitamin D_2_, which subsequently converts to vitamin D_2_ via thermal isomerization (Figure 1). This process forms the basis of mushroom biofortification, where UV-treated mushrooms are enhanced with vitamin D_2_ content using standardized irradiation protocols (e.g., pulsed UV-B lamps or continuous UV chambers). Such an approach offers a plant-based, eco-friendly method to address micronutrient deficiencies [34]. Once ingested as part of UV-treated mushroom-based foods or supplements, vitamin D_2_ is absorbed in the small intestine through passive diffusion and facilitated by bile-salt micelles, a process enhanced in the presence of dietary fats due to micelle formation. After absorption, it is transported to the liver, where CYP2R1 hydroxylates it to form 25-hydroxyvitamin D_2_ [25(OH)D_2_], the major circulating metabolite. The kidneys further hydroxylate this compound via CYP27B1, producing the biologically active form, 1,25-dihydroxyvitamin D_2_ [1,25(OH)_2_D_2_]. This active metabolite binds to the nuclear vitamin D receptor (VDRs), regulating gene expression involved in calcium and phosphorus homeostasis, skeletal health, and modulation of innate and adaptive immune pathways [30]. Thus, the conversion of ergosterol to vitamin D_2_ through UV-based biofortification provides a sustainable dietary strategy to alleviate vitamin D deficiency, particularly in populations with limited sunlight exposure or inadequate dietary intake [35].

Due to diverse climatic conditions, such as varying altitudes and soil rich in organic carbon in the Himalayas, this environment provides an ideal environment for the growth of unique medicinal and nutraceutical beneficial mushrooms [36]. Environmental factors such as altitude, temperature, and nutrient availability are known to influence sterol metabolism, including ergosterol biosynthesis, with higher altitudes and stronger UV exposure potentially enhancing ergosterol accumulation [37]. However, systematic profiling of ergosterol in Himalayan mushrooms and its correlation with these environmental variables remains limited, highlighting a critical research gap.

This review integrates current scientific evidence on ergosterol resources derived from mushrooms, with a focus on species from the Himalayan region, examining their ergosterol content, biosynthetic capacity, and potential nutraceutical applications. Perspectives are further linked to environmental determinants that may regulate ergosterol levels and, consequently, influence the development of mushroom-based nutraceuticals. By synthesizing this evidence, the review aims to contribute to the literature on bioactive mushroom compounds and provide a foundation for sustainable harvesting, conservation, and the potential development of functional foods and biopharmaceutical products derived from Himalayan mushrooms.

## 2. Literature Search Methodology

Available electronic databases, including Google Scholar, PubMed, Scopus, and Web of Science, were searched for studies related to the occurrence, extraction, bioactivities, and potential applications of ergosterol from Himalayan edible mushrooms. The primary search keywords were (1) Ergosterol (title) and mushroom (topic), and (2) Ergosterol (title) and bioactive compound (topic). Additional search combinations included: (1) Ergosterol (title) and vitamin D_2_ (topic); (2) Ergosterol (title) and biosynthesis (topic); (3) Ergosterol (title) and antioxidant (topic); (4) Ergosterol (title) and antimicrobial (topic); (5) Ergosterol (title) and pharmacological properties (topic); (6) Ergosterol (title) and functional foods (topic); (7) Ergosterol (title) and health benefits (topic); (8) Himalayan mushrooms (title) and diversity (title); and (9) Edible mushroom (title) and functional food (title). Relevant articles published primarily between 2000 and 2025 were considered with particular emphasis on recent contributions made after 2015. A total of 395 articles were retrieved, and after screening for relevance and quality, 202 publications were included and discussed in this review. Peer-reviewed articles, reviews, and book chapters reporting ergosterol content, bioactivity, biosynthesis pathways, and diversity.

## 3. Study of Mushrooms in the Himalayas

### 3.1. Importance of Himalayan Mushrooms

**Figure 2:**
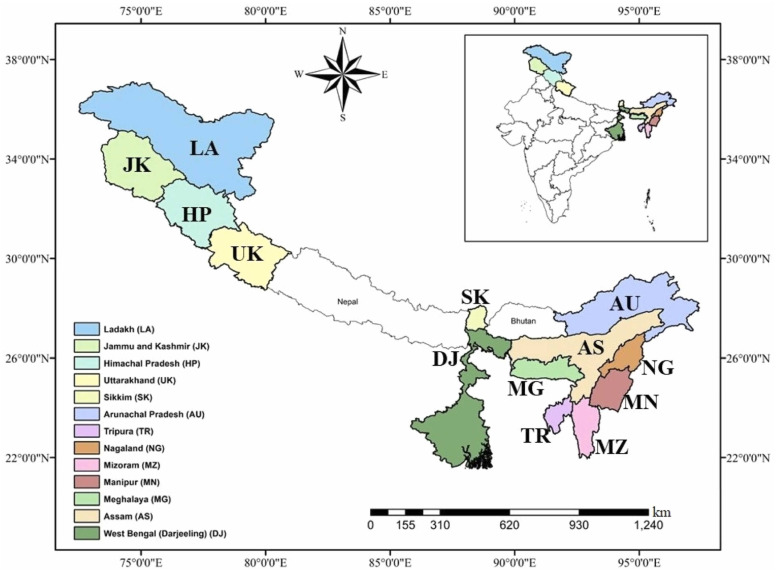
The Indian Himalayan Region (IHR). Reprinted from Ref. [39].

The Himalayan region comprises four parallel mountain ranges: the Great Himalayas (or higher Himalayas), the Lesser Himalayas, the Outer Himalayas (Shivalik Hills), and the Trans Himalayas (including the Tibetan Plateau). The Himalayas stretch across different Asian countries, including India, Nepal, China, Pakistan, and Bhutan, with nearly 55% of its area located within India [38]. The Indian Himalayan region (IHR) encompasses the northern and eastern parts of India. The states of the north and union territories include Jammu and Kashmir, Ladakh, Himachal Pradesh, and Uttarakhand (Figure 2) [39]. The eastern states are Arunachal Pradesh, Manipur, Meghalaya, Nagaland, Sikkim, Mizoram, Tripura, West Bengal, and Assam. The IHR encompasses diverse landscapes and altitudinal gradients ranging from the foothills (~200 m above sea level) to high alpine zones (>6000 m), creating distinct ecological niches that support a rich mushroom diversity [3]. The Himalayas are recognized as one of the global biodiversity hotspots, with mushrooms playing a significant role in ecological processes such as nutrient cycling, organic matter decomposition, and plant symbiosis. Beyond their environmental role, Himalayan mushrooms hold cultural, economic, and medicinal importance, serving as food resources, traditional remedies, and contributors to local livelihoods.

### 3.2. Ecological Significance

Mushrooms are essential to the Himalayan ecosystem, as they play a crucial role in nutrient cycling and maintaining soil fertility. They decompose complex organic matter, releasing bioavailable forms of nitrogen and phosphorus, which are essential macronutrients for plant growth and forest productivity [40]. In addition, many Himalayan mushrooms form ectomycorrhizal associations with dominant tree species such as *Pinus roxburghii* (Chir pine) and *Quercus leucotrichophora* (Banj oak). These associations enhance water and nutrient uptake by trees, while the fungi obtain photosynthetically derived carbohydrates from their hosts, representing a mutualistic interaction [41,42].

Beyond nutrient dynamics, certain mushrooms contribute to forest regeneration by promoting seed germination and seedling establishment. For example, *Tricholoma matsutake* forms characteristic “fairy rings”, which are circular zones of enriched soil with elevated nutrient availability that foster favorable conditions for plant growth [43]. Thus, Himalayan mushrooms function as ecological engineers, influencing nutrient dynamics, plant colonization, and overall forest biodiversity.

### 3.3. Diversity of Wild Edible Macrofungi from the Himalayas

The Himalayas are a fungal hotspot, with over 2000 mushroom species reported, of which approximately 20% are edible. However, only around 60 species have been commercialized at scale [44,45]. The region’s distinct climatic conditions and altitudinal gradients provide a variety of habitats that harbor numerous mushroom species [46,47]. The Himalayas are home to several highly valued edible mushrooms, each with unique characteristics and applications. Some of the most important species are *Morchella* spp., also known as morels, which are the most valuable mushrooms globally. They are recognized for their honeycomb morphology and strong earthy flavor [48]. They are collected in temperate forests during spring and dried for export, fetching premium prices in international markets [49]. *Cordyceps sinensis* (caterpillar fungus) fungus parasitizes caterpillar larvae and grows at high altitudes (3000–5000 m). Traditional medicine greatly prizes it for its energy-enhancing and immune-stimulating activity [50]. *Cordyceps* is a crucial economic harvest in the Himalayas [51]. *Agaricus* spp. (button mushrooms) widely cultivated worldwide, also occurs in Himalayan grasslands and forests, serving as a rich source of protein, vitamins, and minerals [52]. *Cantharellus* spp. (chanterelles) valued for their fruity aroma and mild taste. They are associated with oak and pine trees and are utilized in fine cuisine [53]. *Tricholoma matsutake* (matsutake mushrooms) are of high export value and have annually been imported more than 3000 tons, particularly to East Asian markets such as Japan and Korea, where they are prized for their pungent aroma and firm texture [54,55]. *Ganoderma* spp. (reishi) are renowned for their triterpenoids and polysaccharides with documented immunomodulatory and antioxidative effects, and are commonly consumed as decoctions or standardized extracts [56,57]. Explorations in Sikkim have revealed new edible mushroom species, including *Russula gnathagensis*, *Ramaria thindii*, and *Ramaria subalpina*, the latter traditionally identified and consumed by local communities [58]. Additionally, proximate analysis of *Lentinus squarrosulus*, *Lentinus tuberregium*, and *Macrocybe gigantea* revealed high levels of proteins, dietary fiber, and essential minerals, with *M. gigantea* showing particular potential as an alternative protein source for indigenous populations [59]. Approximately 130 species of edible mushrooms found across the Himalayan region are listed in Table 1.

The Himalayas harbor several endemic and rare mushroom species [3]. However, most of these species are endangered by habitat loss, overharvesting, and climate change [80]. Conservation measures are crucial to safeguard these precious resources [81]. For instance, overexploitation of *Cordyceps sinensis* has resulted in population declines of up to 70% in parts of Nepal and India over the past two decades [82,83]. Despite government regulations restricting unauthorized harvesting and trade, illegal collection continues, with seizures reported in Uttarakhand and Nepal indicating persistent demand and high black-market value [84]. The fruiting season of Himalayan mushrooms is strongly influenced by environmental conditions: morels and *Cordyceps* appear in spring, whereas chanterelles and matsutake emerge in late summer and monsoon periods. Fruiting initiation is regulated by soil moisture and temperature thresholds, making its distribution highly sensitive to climate variability [85,86].

Habitat loss due to deforestation, agricultural expansion, and urban encroachment has reduced fungal richness, particularly ectomycorrhizal taxa dependent on oak and conifer hosts [87]. Habitat conservation and sustainable harvesting are crucial for preserving this biodiversity [88,89]. Community-based management schemes have also been applied in Nepal to organize the collection of *Cordyceps sinensis* [90]. Monitoring and conservation processes are carried out through the engagement of residents, thereby ensuring the commodity is collected sustainably. In the Indian Himalayas, farmers have adopted *Agaricus bisporus* (button mushroom) cultivation as a supplementary livelihood option, reducing dependence on wild harvesting [91]. This enhanced livelihoods and helped mitigate pressure on wild mushroom resources. In Bhutanese traditional medicine, *Ganoderma lucidum* is widely used for its therapeutic properties to treat numerous ailments [92]. Indigenous practitioners have retained knowledge of its healing properties, which have been validated by pharmacological studies demonstrating antioxidant, hepatoprotective, and immunomodulatory effects of *Ganoderma lucidum* [93].

## 4. Ergosterol Biosynthesis in Mushrooms

### 4.1. Egrostrol Structure and Function

**Figure 3:**
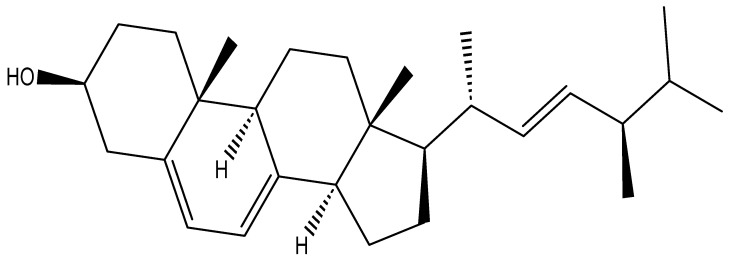
Chemical structure of ergosterol ((22E)-Ergosta-5,7,22-trien-3β-ol).

Ergosterol (ergosta-5,7,22-trien-3β-ol) is a type of sterol in fungi and yeasts, a steroid structure with four fused hydrocarbon rings, double bonds at the C5-C6, C7-C8, and C22-C23 positions, and a 3-β-hydroxy group or 5,7-diene oxysterol, as shown in Figure 3 [94]. It functions analogously to cholesterol in animal cell membranes by regulating membrane fluidity, permeability, and structural integrity [87]. Beyond maintaining cell membrane structure, ergosterol is involved in intracellular signaling, vesicle formation, and endocytosis, thereby contributing to membrane trafficking processes [95]. Moreover, ergosterol and its derivatives, such as ergosterol peroxide, have been reported to exhibit antifungal, antimicrobial, and cytotoxic properties, inhibiting the growth of competing fungi and pathogenic microorganisms [96]. For example, a study conducted by Daroodi et al. [97] found that *Acrophialophora jodhpurensis* produced antifungal metabolites, ergosterol peroxide, capable of controlling destructive rhizoctonia diseases commonly found in tomato.

### 4.2. Biosynthesis of Ergosterol

Ergosterol biosynthesis is a multistep and energy-intensive process that involves more than 20 enzymes across the cytosol and endoplasmic reticulum. The regulation of ergosterol synthesis includes several overlapping mechanisms. These mechanisms involve the expression of the enzyme, feedback inhibition, and shifts in subcellular localization. The process itself involves different enzymes and multiple intricate steps [98,99]. The pathway is not only crucial for the structural importance of ergosterol in fungal cell membranes, but also for the precursor of various secondary metabolites with significant biological functions [100]. Beyond its structural role in fungal membranes, ergosterol serves as a precursor for bioactive secondary metabolites with antifungal, immunomodulatory, and pharmacological properties [101]. The pathway consumes adenosine triphosphate (ATP) and nicotinamide adenine dinucleotide phosphate (NADPH) at several phosphorylation (e.g., mevalonate to mevalonate-5-phosphate) and reduction steps (e.g., 3-hydroxy-3-methylglutaryl coenzyme A (HMG-CoA) to mevalonate), underscoring its high energetic cost.

**Figure 4:**
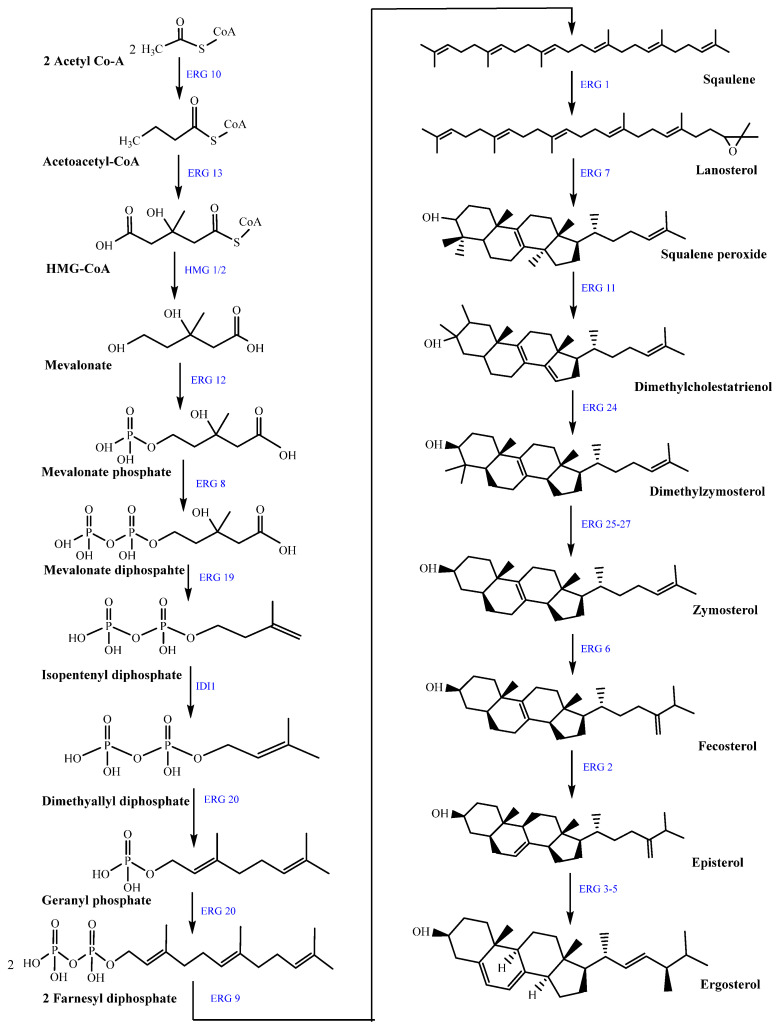
Ergosterol biosynthetic pathway in fungi. The pathway begins with acetyl-CoA and proceeds via the mevalonate pathway to generate isoprenoid intermediates (mevalonate, IPP, FPP), followed by conversion to squalene, lanosterol, zymosterol, and ultimately ergosterol. Blue labels represent ERG genes encoding enzymes that catalyze each step (e.g., ERG1—squalene epoxidase, ERG11—lanosterol 14α-demethylase, ERG6—sterol C-24 methyltransferase). This pathway is essential for fungal membrane integrity and is a major target for antifungal drugs (e.g., azoles inhibit ERG11, allylamines inhibit ERG1).

As shown in Figure 4, the biosynthetic route is commonly divided into three functional segments: the mevalonate pathway for generating isoprene units, the isoprenoid pathway for chain elongation, and the sterol biosynthesis pathway for final sterol modifications.

The pathway begins in the mevalonate branch, where two acetyl-CoA molecules are condensed into acetoacetyl-CoA by acetyl-CoA C-acetyltransferase (ERG10, a thiolase enzyme), followed by conversion to HMG-CoA by HMG-CoA synthase (ERG13). The reduction of HMG-CoA to mevalonate, catalyzed by HMG-CoA reductase (HMG1/2, a class I reductase enzyme), represents the key rate-limiting step in sterol biosynthesis [102].

In the mevalonate pathway, mevalonate undergoes sequential phosphorylation by mevalonate kinase (ERG8) and phosphomevalonate kinase (ERG19), followed by decarboxylation to yield isopentenyl diphosphate (IPP). Isomerization by IDI1 produces dimethylallyl diphosphate (DMAPP), which is condensed by prenyltransferases such as farnesyl pyrophosphate synthase (ERG20) to generate GPP and FPP. Two FPP molecules are then joined by squalene synthase (ERG9) to form squalene. In the sterol biosynthetic branch, squalene is oxidized to 2,3-oxidosqualene by squalene epoxidase (ERG1, a flavoprotein monooxygenase) and cyclized to lanosterol by lanosterol synthase (ERG7, an oxidosqualene cyclase). Lanosterol then undergoes sequential reactions including demethylation (ERG11, CYP51A1, a cytochrome P450 monooxygenase), reductions (ERG24, ERG25–27), and isomerizations (ERG2, C-8 sterol isomerase). These steps yield intermediates such as zymosterol, fecosterol, and episterol. Further desaturation by C-5 sterol desaturase (ERG3) and C-22 desaturase (ERG5), followed by C-24 reduction (ERG4), completes the biosynthetic network leading to ergosterol [26,102,103,104].

### 4.3. Physiological Role of Ergosterol in Fungal Cell Membranes

**Figure 5:**
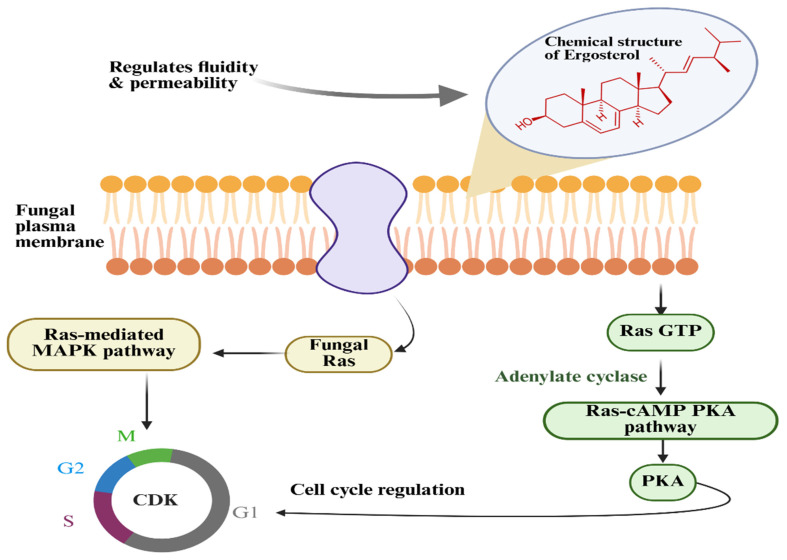
Physiological role of ergosterol in fungal plasma membrane signaling and cell cycle regulation. Abbreviations: cAMP: cyclic adenosine; CDK: cyclin-dependent kinase; GTP: guanosine triphosphate monophosphate (cAMP); MAPK: Mitogen-Activated Protein Kinase; PKA: protein kinase A.

Sterols in fungi, particularly ergosterol, are integral to the lipid bilayer structure, where they regulate growth, survival, and adaptation to environmental stresses by modulating membrane fluidity, phase separation, and permeability through specific sterol-lipid interactions [25]. Ergosterol is also implicated in signal transduction pathways and cell cycle regulation, beyond its structural role [105]. It maintains the dynamic fluidity range of fungal membranes, ensuring the proper folding and activity of membrane proteins, such as nutrient transporters, ATP-binding cassette (ABC) efflux pumps, and G–protein–coupled receptors [106]. Ergosterol within the membrane also affects its permeability, preventing leakage of ions and other small molecules. Ergosterol plays a part in the typical function of membrane-bound proteins such as transporters, receptors, and enzymes (Figure 5). It plays a role in the function of such proteins by altering their conformation and interactions with other membrane proteins [107]. It is further involved in signal transduction cascades, particularly the Ras-cyclic adenosine (cAMP)-protein kinase A (PKA) pathway, which regulates cell proliferation, differentiation, and stress response [108,109]. Moreover, ergosterol-mediated modulation of cyclin-dependent kinases (CDKs) has been reported to influence cell cycle progression, linking sterol metabolism to fundamental regulatory checkpoints [110].

### 4.4. Influence of Environmental Factors on Ergosterol Production

Environmental parameters such as oxygen availability, pH, temperature, and nutrient supply strongly modulate ergosterol biosynthesis in fungi by altering both ERG gene expression and enzyme activity in the mevalonate and sterol branches of the pathway [111,112].

Temperature is among the most essential factors influencing ergosterol production by fungi [113]. In *Saccharomyces cerevisiae*, ergosterol synthesis peaks at 30 °C, which corresponds to the optimal growth curve of the organism. At 15 °C, the expression of ERG11 and ERG25 drops by >40%, reducing ergosterol levels by ~35% compared to 30 °C, whereas at 37 °C, the accumulation of sterol intermediates such as lanosterol indicates pathway disruption [98,114].

The pH of the growth medium can also affect the production of ergosterol in fungi. Fungi produce maximum ergosterol accumulation at pH 6.0 (~60% higher than at pH 4.0/8.0) under neutral to slightly acidic pH [115]. Extreme levels of pH (either acidic or alkaline) would inhibit the activities of enzymes in the ergosterol biosynthesis pathway. Therefore, there would be a lesser production of ergosterol [116]. Arthington-Skaggs et al. [117] investigated the impact of pH on ergosterol production in the human pathogenic fungus *Candida albicans* and suggested that the optimal ergosterol content was at a pH of 6.0, which is near the favorable pH for the growth of *C. albicans*. In this study, the ergosterol content decreased approximately 60% at pH 4.0 and 70% at pH 8.0, along with the suppression of the enzymatic activity necessary for ergosterol biosynthesis. This research thus indicates that the regulation of ergosterol production in fungi is significantly influenced by pH.

In the ergosterol biosynthesis pathway, oxygen is required in the oxidation of squalene to 2,3-oxidosqualene. Tan et al. [118] found that the availability of nutrients further modulates sterol synthesis. Carbon excess, especially glucose, upregulates ERG9 (squalene synthase) and ERG11, increasing ergosterol by 2–3 fold [119]. Conversely, nitrogen starvation downregulates ERG2 and ERG6, reducing ergosterol by ~40% and compromising membrane fluidity and stress tolerance [120].

Together, these factors demonstrate that ergosterol biosynthesis is a multi-enzyme, multi-intermediate process tightly linked to environmental conditions. These insights are directly relevant to antifungal drug design, azoles and allylamines target oxygen-dependent enzymes (e.g., ERG11, ERG1), making environmental modulation of ergosterol a key determinant of drug susceptibility [26]. The role of environmental regulation in ergosterol production by fungi provides valuable insights into the physiological function of ergosterol in fungal cell membranes.

## 5. Ergosterol and Vitamin D_2_ Conversion

### 5.1. Ergosterol and Vitamin D_2_ Contents of the Mushrooms

Ergosterol, the dominant sterol present in mushrooms, undergoes photochemical conversion upon UV-C (100–280 nm) or UV-B (280–320 nm) irradiation through a [6π] electrocyclic ring-opening reaction, producing pre-vitamin D_2_, which thermally isomerizes to vitamin D_2_. However, the efficiency of this process depends on irradiation parameters such as wavelength, intensity (0.6–1.2 W/m^2^), and exposure time (40–120 min), as shown in experimental optimization studies [121,122]. Huang et al. [123] recorded 1.41 mg/g of ergosterol in fresh shaggy mushrooms, which was changed to 1.17, 0.88, and 1.43 mg/g after UV-irradiation for 60, 30, and 10 min, respectively. Similarly, in this study, 2.29 mg/g of ergosterol was documented in the oyster mushrooms, which was changed to 2.05, 2.37, and 2.29 mg/g after UV-irradiation for 60, 30, and 10 min, respectively. In another study, the highest vitamin D_2_ levels of 0.95–1.03 mg/g dry weight (DW) were obtained with an intensity of 0.31 mW/cm^2^ for 10 min in *A. bisporus* [33]. Ergosterol and vitamin D_2_ content in diverse cultivated and wild mushrooms are provided in Table 2.

Kristensen et al. [124] demonstrated that mushrooms can achieve a specific content of vitamin D_2_ when exposed to extra UV-B radiation just before harvest, increasing from 0.2 μg/100 g to 11.6 μg/100 g. In terms of ergosterol reservoirs, cultivated mushrooms exhibited higher sterol concentrations than wild species from Serbia and Korea [125]. Sun et al. [33] recorded ergosterol levels ranging from 2290 to 6200 μg/g, with the highest in *Agaricus bisporus*, followed by *Lentinula edodes* and *Pleurotus ostreatus*. Among medicinal taxa, *Pleurotus* sp. contained 64.56 mg/100 g DW, while in vitro cultures of *Pleurotus djamor* under glucose-supplemented, aerated liquid culture conditions produced 37.96 mg/100 g DW(37.96 mg/100 g DW) [126]. Ergosterol and vitamin D_2_ content in diverse cultivated and wild mushrooms are listed in Table 2.

**Table 2:** Ergosterol and vitamin D_2_ content in diverse cultivated and wild mushrooms.

Mushroom Species	Mode of Production	Ergosterol (mg/g)	Vitamin-D_2_ (µg/g)	Reference
*Agaricus bisporus*	Cultivated	0.532–0.598	0.07–0.23	[127]
*Flammulina veluptipes*	Cultivated	0.299–0.446	0.04–0.40	
*Lentinus edodes*	Cultivated	0.744–1.07	0.03–1.15	
*Grifola frondosa*	Cultivated	0.281–1.06	0.08–0.12	
*Pleurotus ostreatus*	Cultivated	0.567–0.773	0.07–2.59	
*Agaricus bisporus*	Cultivated	0.533–0.685	0.03–0.08	
*Agaricus bisporus*	Cultivated	0.539–0.681	0.05–0.77	
*Agaricus bisporus*	Cultivated, *UV-treated*	0.422–0.606	3.36–20.9	
*Cantharellus* sp.	Cultivated	0.463–0.677	2.18–8.41	
*Morchella* sp.	Cultivated	0.207–0.326	4.39–6.26	
*Agaricus subrufescens*	Cultivated	1.66	11.43	[123]
*Agrocybe aegirit*	Cultivated	3.49	13.75	
*Armillaria mellea*	Cultivated	1.92	14.78	
*Auricularia auricula*	Cultivated	3.01	11.06	
*Boletus aereus*	Cultivated	0.64	15.46	
*Boletus aereus*	Wild growing	4.69	14.94	
*Boletus luridus*	Cultivated	4.15	15.91	
*Boletus pinophilus*	Cultivated	2.17	23.68	
*Cantharellus cibarius*	Wild growing	3.03	20.61	
*Chroogomphis rutillus*	Wild growing	1.55	10.34	
*Collybia albuminosa*	Wild growing	3.06	21.92	
*Coprinus comatus*	Cultivated	1.13	15.79	
*Cordyceps militaris*	Cultivated	1.43	7.67	
*Ganoderma lucidum*	Cultivated	1.93	14.43	
*Griflola frondosa*	Cultivated	1.64	15.95	
*Hellinus igniarius*	Wild growing	1.03	15.69	
*Hericium erinaceus*	Cultivated	0.023	11.32	
*Hohenbuehelia serotina*	Cultivated	2.77	24.1	
*Hypsizygus marmoreus*	Cultivated	1.58	25.02	
*Pleurotus citrinopileatus*	Wild growing	2.17	12.67	
*Lentinus edodes*	Cultivated, processed	3.06	24.73	
*Lentinus edodes*	Cultivated	0.93	17.16	
*Morehella esculenta*	Wild growing	0.27	12.7	
*Phallus indusiatus*	Cultivated	1.5	15.43	
*Pholiota namek*	Cultivated	2.3	19.52	
*Poria cocos*	Cultivated	2.24	14.51	
*Ramaria botrytoides*	Wild growing	1.38	24.05	
*Russula Virescens*	Cultivated	1.1	15.08	
*Suillus bovinus*	Cultivated	2	24.63	
*Tremella fuciformis*	Cultivated	0.81	12.55	
*Tremella mesentarica*	Wild growing	1.13	17.71	
*Tricholoma matsutake*	Wild growing	3.16	21.6	
*Tricholoma mongolicum*	Cultivated	2.75	24.37	
*Tuber melanosporum*	Wild growing	1.08	15.35	
*Volvariella volvacea*	Cultivated	2.03	2.03	
*Pleurotus ostreatus*	Cultivated, UV-C treated	1.41	67	
Shaggy ink cap	Cultivated, UV-C treated	2.29	229.7	
Shiitake	Cultivated	0.0605	-	[128]
Enoki	Cultivated	0.0068	-	
Button	Cultivated	0.078	-	
Oyster	Cultivated	0.044	-	
Abalone	Cultivated	0.0435	-	
*A. arvensis* (Mycelia M7400)	Cultivated	0.0268	-	[129]
*A. bisporus* (white) (Amycel 2600)	Cultivated	0.217	-	
*A. bisporus* (brown) (Hollander Spawn C9)	Cultivated	0.264	-	
*A. bisporus* (white) (Sylvan 767)	Cultivated	0.095	-	
*A. bisporus* (white) (Italspawn F599)	Cultivated	0.04	-	
*A. bisporus* (white) (Kanmycel 3-1)	Cultivated	0.361	-	
*A. bisporus* (white) (Kanmycel K2)	Cultivated	0.184	-	
*A. bisporus* (white) (Sylvan A15)	Cultivated	0.011	-	
*A. brasiliensis*	Cultivated	0.06	-	
*A. bitorquis*	Wild growing	0.355	-	
*A. silvaticus*	Wild growing	0.458	-	
*A. campestris*	Wild growing	0.424	-	
*A. bisporus*	Soil growing	0.246	-	
*C. cibarius*	Soil growing	0.017	-	
*C. gigantea*	Soil growing	0.159	-	[130]
*L. fumosum*	Soil growing	0.15	-	
*L. gilva*	Soil growing	0.145	-	
*L. scabrum*	Soil growing	0.133	-	
*M. esculenta*	Soil growing	0.122	-	
*M. procera*	Soil growing	0.168	-	
*S. crispa*	Soil growing	0.007	-	
*S. bovinus*	Soil growing	0.082	-	
*T. equestre*	Wood growing	0.107	-	
*A. mellea*	Wood growing	0.113	-	
*A. auricula-judae*	Wood growing	0.11	-	
*F. hepatica*	Wood growing	0.237	-	
*G. frondosa*	Wood growing	0.079	-	
*L. sulphureus*	Wood growing	0.54	-	
*P. squamosus*	Wood growing	0.211	-	
*Lentinula edodes* (Shiitake)	Cultivated	5.158	-	
*Lentinula edodes* (chuje 2)	Cultivated	4.718	-	
*Pleurotus pulmonarius* (Santali)	Cultivated	4.337	-	[131]
*Flammulina velutipes* (Megumi’ Enoki)	Cultivated	2.656	-	
*Hericium erinaceus* (Nolu)	Cultivated	3.86	-	
*Auricularia polytricha* (Wood ear)	Cultivated	0.82	-	
*Agaricus bisporus*	Cultivated	7.762	-	
*Pleurotus eryngii*	Cultivated	3.052	-	
*Pleurotus eryngii* var. ferulae	Cultivated	2.29	-	
*Hypsizigus marmoreus* (Haemi)	Cultivated	4.27	-	
*Hypsizigus marmoreus* (Baegman 1)	Cultivated	1.51	-	
*Pleurotus ostratus* (Jacq.) Kummer (Konji 7)	Cultivated	6.26	-	
*Pleurotus ostratus* (Jacq.) Kummer (Sunjung)	Cultivated	5.37	-	
*Pleurotus ostratus* (Jacq.) Kummer (Baekseon)	Cultivated	7.624	-	
*Agaricus bisporus* (white)	Cultivated	6.54	-	
*A. bisporus* (brown)	Cultivated	6.02	-	
*Pleurotus ostreatus*	Cultivated	6.74	-	[132]
*Lentinus edodes*	Cultivated	6.79	-	
*Chantarellus cibarius*	Wild growing	3.04	0.84	
*Chantarellus tubaeformis*	Wild growing	3.77	1.94	
*Boletus edulis*	Wild growing	4.89	0.047	
*Lactarius trivialis*	Wild growing	2.96	0.29	
*Cantharellus tubaeformis*	Wild growing	0.168	0.211	
*Cantharellus cibarius*	Wild growing	0.24	0.1	
*Boletus edulis*	Wild growing	1.922	0.58	[133]
*Agaricus bisporus* (white)	Dark cultivated	0.44	0.006	
*Agaricus bisporus* (brown)	Dark cultivated	0.39	0.003	
*Agaricus bisporus* (Portabella)	Dark cultivated	0.56	0.008	
*Lentinus edodes*	Dark cultivated	1.07	0.012	
*Pleurotus ostreatus*	Dark cultivated	0.607	0.007	
*Cantharellus cibarius*	Canned mushrooms	0.1	0.12	
*Agaricus bisporus* (white)	Canned mushrooms	0.13	0.006	
*Cantharellus tubaeformis*	Dark cultivated, UV-C treated	0.57	14.03	
*Agaricus bisporus* (white)	Wild growing, UV-C treated	4.53	10.14	

### 5.2. Factors Affecting Ergosterol and Vitamin D_2_ Contents

Several factors influence the conversion of ergosterol into vitamin D_2_ in mushrooms, including material form, wavelength, exposure time, and irradiation conditions. These factors can be employed to increase vitamin D_2_ content in mushrooms. For instance, controlled exposure to direct sunlight has been shown to elevate vitamin D_2_ levels, although, limited by natural variation. This technique is inexpensive but less controlled, as the sun’s intensity varies with the weather and location. Phillips et al. [134] found that in white button mushrooms, 15 min of sunlight exposure on a partly cloudy and clear day can increase vitamin-D_2_ by 157–754 IU per 70 g serving (26–126% of the Dietary Reference Intake, DRI) while 30 min can result in increases of 1142 IU per 70 g serving (>100% DRI). Overall, among all the studied mushrooms, 15 min of exposure can increase vitamin D_2_ by 76–178 IU per 70 g serving (13–30% of the DRI), and 60 min of exposure can achieve levels comparable to those achieved in 15 min under clear conditions. Oyster and enoki mushrooms demonstrated higher conversion efficiencies, with vitamin D_2_ levels reaching 832–886 IU per 70 g serving after 15 min of sunlight exposure.

UV-B treatment significantly increased the vitamin-D_2_ content in the edible and by-products of mushrooms [135]. Vitamin D_2_ content of 2.8, 13.8, 40.7, and 61.9 µg/g DW was recorded in whole shiitake mushrooms exposed to 0, 25, 50, and 75 kJ/m^2^ of UV-B radiation at 25 °C, respectively [136]. Similarly, UV-B exposure, at a dose of 25 kJ/m^2^, increased the concentration of vitamin-D_2_ to 36.7, 68.6, and 106.4 µg/g DW for the middle, pileus, and gill parts of the shiitake mushroom, respectively. When white button mushrooms were sliced before irradiation, the increase was more pronounced due to the greater exposed surface area. Wu et al. [137] also identified UV-B as the most efficient irradiation source, with a temperature of 25–45 °C, an exposure time of 40–120 min, and an irradiation intensity of 0.6–1.2 W/m^2^. Notably, slicing mushrooms before UV-B exposure resulted in vitamin D_2_ contents up to 406 μg/g, representing approximately a tenfold increase relative to whole mushrooms irradiated under similar conditions [138]. These studies demonstrated the effectiveness of UV-B treatment in increasing vitamin D concentration.

The drying methods and drying temperature also affect the vitamin D_2_ content in dehydrated mushrooms. Nolle et al. [139] recorded maximum concentrations of 171.84 µg/g (freeze-dried shiitake) and 169 µg/g (hot-air dried shiitake at 60 °C for 180 min), though sample sizes were not reported. In contrast, the lowest amount of 34.56 µg/g was observed in brown button mushrooms dehydrated at 40 °C for 415 min.

Ergosterol is a key building block in the synthesis of vitamin D_2_. When mushrooms are exposed to UV light, whether from natural sources or artificial ones, their vitamin D_2_ content can increase significantly. This makes them an essential source of the vitamin in the diet. Methods for enriching vitamin D_2_ have been developed and are commonly used in both research and commercial settings [140], but further work is needed to quantify conversion efficiencies across species, establish reproducible baselines, and evaluate the potential of medicinal mushrooms as optimized sources of dietary vitamin D_2_.

## 6. Impact of Himalayan Environmental Factors on Ergosterol Content

The Himalayan region, characterized by its distinct environmental conditions, including high altitude, extreme UV radiation, and varied soil composition, provides a unique natural setting for studying ergosterol biosynthesis and accumulation in mushrooms [44]. The environmental conditions significantly affect the metabolic processes of fungi, such as the biosynthesis of ergosterol, an essential part of fungal cell membranes and a precursor to vitamin D_2_ [141].

### 6.1. Role of Altitude, UV Exposure, and Soil Composition in Ergosterol Biosynthesis

Decreased atmospheric pressure results in lower oxygen tensions at elevated altitudes, which affects the biodiversity of mushrooms [142]. Oxygen is also essential for key reactions in the ergosterol pathway, such as the epoxidation of squalene to 2,3-oxidosqualene, catalyzed by squalene epoxidase (ERG1) [98]. High-altitude areas show extreme temperature variation between day and night [143]. Fluctuations could affect the catalytic activity of enzymes involved in ergosterol biosynthesis and, thus, production [98]. Similarly, the Himalayan region receives increased amounts of UV radiation due to its higher elevations and thinner atmosphere [144]. Comparative studies of wavelength effects have shown that UV-A (315–400 nm), UV-C (100–280 nm), or their combination induces stronger effects on fungal metabolism than UV-B (280–315 nm) alone [145]. UV radiation, especially UV-B, has been shown to facilitate the conversion of ergosterol to vitamin D_2_ [146]. At the same time, higher doses of UV might also injure fungal cells and result in adaptive processes that would interfere with ergosterol biosynthesis [147]. Such adaptations may include the upregulation of ergosterol production to stabilize cell membranes and protect against oxidative stress [148].

The soil in the Himalayas is often rich in organic matter, resulting from the decomposition of plant material, which provides essential nutrients for fungal growth [149]. In edible mushrooms from Serbia and Korea, the levels of zinc, copper, and iron differed among mushroom species. In a comparative study of edible mushrooms from Serbia and Korea, 13.1–149.7 mg/kg of zinc, 8.5–479.9 mg/kg of copper, and 1.62–93.03 mg/kg of iron were reported [125]. In this study, the principal component analysis (PCA) revealed that iron levels correlated positively with ergosterol content, with two extracted components explaining 79.14% of the total variance. The Mg and Zn as enzyme cofactors (sterol C-14 reductase, sterol methyltransferase) of the soil can vary significantly, which affects the availability of essential elements such as magnesium and zinc. These elements act as cofactors for enzymes in the ergosterol biosynthesis pathway [125]. The acidic nature and moisture-retention properties of Himalayan soils can influence fungal growth and ergosterol production [150]. Fungi may adapt to these conditions by favoring ergosterol accumulation under stress [151].

### 6.2. Comparison of Ergosterol Levels in High-Altitude vs. Low-Altitude Mushrooms

Recent research has reported that mushrooms from high-altitude regions, such as the Kargil area of the Northwest Himalayas, contain ergosterol levels exceeding the typical range documented for many edible mushrooms (0.2–7.8 mg/g DW) [75]. These observations suggest that environmental factors associated with high-altitude habitats may contribute to elevated ergosterol levels in these fungi. Environmental stressors prevalent at high altitudes, such as increased UV radiation, temperature fluctuations, and lower oxygen levels, may stimulate ergosterol biosynthesis in mushrooms as a protective mechanism [152]. These stressors can induce the production of ergosterol, which helps maintain cell membrane stability and protects against oxidative damage [99]. Consequently, mushrooms from high-altitude regions may have a higher ergosterol content, thereby improving their capacity for vitamin D_2_ synthesis upon UV exposure [153]. *Cordyceps sinensis* (*Ophiocordyceps sinensis*) is predominantly found in the high-altitude regions of the Tibetan Plateau, typically between 3000 and 5000 m above sea level [154]. It has been found to contain measurable amounts of ergosterol in cultivated samples, with ergosterol concentrations reaching 555 mg/100 g DW [155], although the percentage of sterol relative to total lipids was not reported. Similarly, morel mushrooms (*Morchella esculenta*) from the Himalayan foothills (2000–3000 m) showed ergosterol levels of 49.175 mg/100 g DW and vitamin D_2_ concentrations of 0.675 mg/100 g DW, values higher than those typically observed in low-altitude counterparts (<20 mg/100 g DW for ergosterol, <0.2 mg/100 g DW for vitamin D_2_) [156]. UV light and nutrient supply in the foothills facilitate the biosynthesis of ergosterol. Case studies of specific Himalayan areas, such as the Tibetan Plateau and the Eastern Himalayas, highlight the adaptive processes of fungi in extreme environmental conditions and their potential as a valuable source of ergosterol and vitamin D_2_ [157]. Future studies should focus on quantifying sterol biosynthesis pathways under defined environmental gradients and evaluating their nutritional and pharmaceutical potential.

## 7. Nutraceutical and Health Benefits of Ergosterol

In addition to its structural function, ergosterol has attracted interest as a nutraceutical and pharmaceutical compound with antioxidant, anti-inflammatory, and possibly therapeutic effects. The health benefits of ergosterol and its status as a bioactive compound are discussed in this section.

### 7.1. Nutraceutical Properties

Ergosterol possesses several nutraceutical properties, including a precursor to vitamin D_2_, which is essential for maintaining bone health, promoting calcium absorption, and supporting the immune system [158]. Moreover, it exhibits antioxidant and anti-inflammatory properties [159].

Correa et al. [160] found that the incorporation of *Agaricus blazei* extract, rich in ergosterol, significantly enhanced the antioxidant capacity of fortified yogurts. This improvement was confirmed through both the reducing power and DPPH assays, with EC_50_ values of 4.59 mg/mL and 59.12 mg/mL, respectively. In another study, Heleno et al. [161] reported a higher EC_50_ value (93 mg/mL) for yogurt supplemented with *Agaricus bisporus* mycosterol extract, indicating a comparatively lower antioxidant potential. Furthermore, the antioxidant capacity of the *A. blazei*-fortified yogurt increased progressively during storage, exhibiting an approximate rise of 17% in the reducing power assay and 12% in the DPPH assay, whereas non-fortified samples showed a gradual decline. These findings suggest that ergosterol and related sterol compounds not only enhance the nutritional quality of foods but also contribute to their functional stability over time.

Kuwabara et al. [162] investigated the effects of daily high ergosterol intake on cholesterol and vitamin D biosynthetic pathways in ovariectomized (OVX) rats, a model for postmenopausal estrogen deficiency. After 14 weeks of ergosterol supplementation, the OVX rats exhibited a significant decrease in plasma cholesterol and sitosterol levels, accompanied by a notable increase in 7-dehydrocholesterol and a slight elevation in 1α,25-dihydroxyvitamin D_3_ concentrations. Importantly, ergosterol intake improved bone strength and reduced bone resorption markers such as serum tartrate-resistant acid phosphatase 5b (TRAP-5b), indicating protection against OVX-induced bone loss. These findings suggest that ergosterol not only enhances the nutritional and antioxidant properties of food matrices but also plays a crucial physiological role in regulating lipid metabolism, vitamin D synthesis, and bone health, thereby underscoring its potential as a functional dietary sterol [162].

### 7.2. Antioxidant and Anti-Inflammatory Properties of Ergosterol

Ergosterol has demonstrated strong antioxidant and anti-inflammatory properties, making it a promising nutraceutical compound [159]. These activities are generally attributed to its ability to scavenge free radicals, regulate inflammatory mediators, and reduce cellular oxidative damage under experimental conditions.

Ergosterol can scavenge reactive oxygen species (ROS) and prevent oxidative cell damage [163]. This helps explicitly guard against chronic disorders like heart disease, neurodegenerative conditions, and cancer [164]. Stastny et al. [165] demonstrated that methanol extracts of *Pleurotus flabellatus* exhibited the highest oxygen radical absorbance capacity (ORAC) within the genus *Pleurotus* (reported as approximately 63.9 mg of Trolox equivalents/g extract), and these extracts contained comparatively high levels of ergosterol (214.5 mg/kg), ergothionine (6.22 mg/g), and mannitol (144.3 mg/g). In this study, the chloroform extract also showed substantial anti-inflammatory cyclooxygenase-2 (COX-2) activity, as assessed using the in vitro enzymatic assay. Similarly, in this study, the 80% methanol extract of *Pleurotus ostreatus* Florida showed the highest inhibition of nuclear factor kappa B (NF-κB) in the human monocytic THP-1 cell line model.

Ergosterol-rich *Hericium erinaceus* ethanolic extract has been shown to inhibit pro-inflammatory cytokine production, including tumor necrosis factor-alpha (TNF-α), interleukin-6 (IL-6), and interleukin-1β (IL-1β) in LPS-stimulated THP-1 monocytic cells [166]. Similarly, in another study, ergosterol inhibited the NF-κB signaling pathway in LPS-induced neuroinflammation in microglia cells and ICR mice, a central controller of inflammation, thus inhibiting inflammation at the molecular level [167]. Zhou et al. [168] demonstrated that the sterols from *Ganoderma lucidum* bind to the active sites of p38 and p65, thereby suppressing their activation. Ergosterol from *G. lucidum* (Reishi) has been shown to significantly reduce inflammation in animal models by inhibiting the NF-κB pathway and lowering levels of pro-inflammatory cytokines.

These findings collectively suggest that ergosterol plays a functional role in modulating oxidative and inflammatory pathways, although more quantitative and clinical evidence is needed to validate its nutraceutical potential.

### 7.3. Potential Pharmacological Applications

Ergosterol’s bioactive properties have been studied for various pharmacological applications, including cancer treatment, neuroprotection, and immune modulation [169]. Ergosterol triggers apoptosis, also known as programmed cell death, in cancer cells by activating caspases and disrupting the mitochondria [170]. In vitro and in vivo studies revealed that it can prevent the formation of new blood vessels, known as angiogenesis, which supply tumors, limiting their growth and spread. Ergosterol may also activate immune cells, such as macrophages and natural killer (NK) cells, thereby enhancing the body’s immune response to disease and infection [171,172]. Ergosterol derivatives are investigated as vaccine adjuvants to improve immune responses [173].

Ergosterol’s antioxidant properties suggest its potential as a neuroprotective agent against oxidative stress, a significant contributor to neurodegenerative disorders like Alzheimer’s and Parkinson’s diseases [174]. Ergosterol derivatives have demonstrated efficacy in diminishing neuroinflammation by inhibiting microglial activation and reducing the release of pro-inflammatory mediators. Ergosterol derivatives, including ergosterol peroxide, demonstrated significant immunomodulatory effects, such as the modulation of cytokine secretion, the inhibition of apoptosis, and the regulation of critical signaling pathways, including NF-κB, p38/MAPK, and retinoic acid-inducible gene-I (RIG-I) in human alveolar epithelial A549 cells [175]. In a separate study, ergosterol peroxide demonstrated metabolic stability, high-dose tolerability, and tumor growth inhibition, while derivatives with enhanced aqueous solubility maintained selective cytotoxicity against triple-negative breast cancer cells [176]. Moreover, in epithelial kidney LLC-PK1 cells, ergosterol peroxide exhibited antiviral properties against porcine deltacoronavirus (PDCoV) by inhibiting viral attachment and entry, suppressing virus-induced apoptosis, and down-regulating cytokine expression through NF-κB and p38/MAPK pathways [177]. These findings collectively underscore the extensive immunomodulatory and therapeutic potential of ergosterol derivatives, indicating their efficacy in managing inflammation, viral infections, and specific malignancies.

Nilkhet et al. [110] reported that ergosterol exhibits antitumor activity through several defined molecular mechanisms. In breast cancer cell models, ergosterol was shown to inhibit the Wnt/β-catenin signaling pathway by downregulating β-catenin expression and suppressing its nuclear translocation, thereby reducing transcription of downstream oncogenes. In addition, ergosterol disrupted cancer cell metabolism by altering glycolytic flux and mitochondrial activity, which impaired tumor cell growth and survival. These findings provide insight into the mechanistic basis of ergosterol’s antitumor potential in breast cancer; however, clinical investigations are required to validate its therapeutic applicability.

### 7.4. Safety, Dosage, and Bioavailability Considerations of Ergosterol and Vitamin D_2_

Ergosterol and its photoconversion product, vitamin D_2_, have gained significant attention as bioactive components of edible mushrooms and as potential functional foods. Although both compounds are generally regarded as safe, recent preclinical and toxicological data have expanded our understanding of their safety margins and pharmacokinetic behavior [178]. The U.S. Food and Drug Administration (FDA), 2020, classifies vitamin D_2_ mushroom powder as a nutrient supplement in specific food categories as “Generally Recognized as Safe (GRAS)” when consumed within the recommended daily limit of 4000 IU for adults [179]. While ergosterol itself remains poorly soluble, recent advancements have focused on improving the bioavailability and therapeutic potential of ergosterol through novel formulation strategies. Zhang et al. [180] developed ergosterol-loaded poly(lactide-co-glycolide) (PLGA) nanoparticles, which demonstrated significantly enhanced oral bioavailability and in vitro antitumor activity compared to free ergosterol. The nanoparticle system provided a controlled release profile and improved stability in simulated gastrointestinal conditions. These findings highlight the potential of nanocarrier-mediated delivery systems in overcoming ergosterol’s poor solubility and absorption limitations, thereby broadening its applicability as a nutraceutical or therapeutic compound.

Further insights into ergosterol’s metabolic influence were provided by a controlled dietary study in male wild-type mice, where diets were supplemented with 0, 2, or 7 mg of ergosterol per kg for six weeks alongside 25 μg of vitamin D_3_ (per kg diet). Results indicated that mice receiving 7 mg/kg ergosterol exhibited 1.3-, 1.7-, and 1.5-fold higher concentrations of vitamin D_3_ in serum, liver, and kidney, respectively, compared with controls (*p* < 0.05). However, the concentrations of 25-hydroxyvitamin D_3_, 1,25-dihydroxyvitamin D_3_, and 24,25-dihydroxyvitamin D_3_ remained unchanged across groups, suggesting that ergosterol enhanced systemic vitamin D_3_ availability without altering hepatic or renal hydroxylation pathways. Lipid analyses also revealed no significant effects on hepatic cholesterol or triglyceride levels. Complementary HepG2 cell experiments confirmed that ergosterol did not influence the enzymatic conversion of vitamin D_3_ to 25(OH)D_3_. Collectively, these findings indicate that ergosterol can modulate vitamin D_3_ concentrations in vivo without disrupting vitamin D metabolism or lipid homeostasis, supporting its dietary safety [181]. A recent study by Ling et al. [176] evaluated *ergosterol peroxide* (a natural oxidation product of ergosterol) and a series of its synthetic derivatives for their biological activity and safety. In vitro assays using triple-negative breast cancer (TNBC) cell lines (SUM149 and MDA-MB-231) demonstrated that several derivatives of ergosterol peroxide reduce tumor growth at 100 mg/kg BW, and it is well-tolerated at 500 mg/kg beyond their expected therapeutic dosage. Importantly, no hepatotoxicity, cardiotoxicity, or significant CYP450 inhibition was observed at physiologically relevant concentrations.

Future research should focus on long-term clinical trials assessing ergosterol’s tolerability, its optimal dosage in fortified foods, and comparative bioavailability between ergosterol-derived vitamin D_2_ and conventional vitamin D_3_ sources. Integrating such preclinical and nutritional data will advance the development of ergosterol-based functional ingredients that are both safe and physiologically effective.

## 8. Sustainable Cultivation of Himalayan Mushrooms

Mushroom cultivation, particularly in the Himalayan region, has garnered tremendous interest due to its nutritional, medicinal, and economic importance. Sustained cultivation practices have ensured high-quality mushroom production while maintaining ecological balance and promoting local economic growth [6,13,44]. This section examines the current state of mushroom cultivation in the Himalayas, technological advancements, and the environmental and financial benefits of sustainable cultivation practices.

### 8.1. Current Status of Mushroom Cultivation in the Himalayan Region

With its unique climatic conditions and high biodiversity, the Himalayan region is an ideal location for mushroom cultivation [62]. A survey by Singh et al. [182] in the Central Himalayan region, primarily in Uttarakhand, specifically in Chamoli, reveals that off-season mushroom cultivation in polyhouses was most favorable, especially for *Pleurotus* sp., with a yearly income of INR 42,000, adopted by 8 out of 120 families. In the northeastern hill areas of India, mushroom farming, particularly of the *Pleurotus florida* and *Pleurotus pulmonarius* species, has contributed to additional income and improved nutritional requirements in rural areas [183]. The industry, however, remains in its infancy stage, with the small-scale and conventional farming style prevailing throughout.

Most mushroom production in the Himalayas is based on traditional practices, including cultivating mushrooms on logs or in natural forest environments. However, these practices are time-consuming and have low production levels. *Pleurotus* spp. (oyster mushroom), *Agaricus bisporus* (button mushroom) and *Cordyceps sinensis* (caterpillar fungus) are two of the most popular species grown and cultivated in the Himalayas. Mushroom growers in these regions face various challenges, including limited access to advanced cultivation methods, market accessibility issues, and inadequate storage and transportation facilities [39,184,185]. Other challenges that most mushroom growers faced were insect pest attacks and diseases. In Himachal Pradesh, about 50.55 percent of respondents reported these problems, as did those in Uttarakhand [185,186]. Mainly, button and oyster mushrooms have witnessed an increase in cultivation in the Himalayan region, which can thrive easily in the local environment. However, farmers continue to use traditional practices, which restrict their yield and earnings.

One of the most expensive and high-demand mushroom species, *C. sinensis*, has had both positive and negative impacts, significantly transforming the rural economy while also increasing illegal trade [187]. This is because, in 2020, the IUCN listed *C. sinensis* as a vulnerable species, and overexploitation has an impact on the diversity of these mushrooms.

### 8.2. Progress in Cultivation Methodologies for High Ergosterol-Yielding Mushrooms

Researchers and producers are implementing advanced cultivation methodologies to increase the yield of high-ergosterol mushrooms, thereby improving production, quality, and sustainability [139,188]. Advanced cultivation houses employ controlled environments to provide optimal temperatures (20–25 °C) and humidity levels (80–90%) for mushroom development, some of which utilize advanced electronic devices to control the conditions [189]. UV-B and UV-C light are applied to the mushrooms in a controlled manner to enhance the conversion rate of ergosterol to vitamin D_2_, making the mushrooms more nutritious [190]. Rice straw, wheat straw, and sawdust are the materials most commonly used for cultivating mushrooms [191,192]. The substrates are readily available at low costs and are helpful for sustainable farming. Supplementing the substrate with nitrogen-rich additions (e.g., soybean meal) can support the growth of mushrooms and the production of ergosterol [193]. Researchers are developing high-yield strains of mushrooms with increased bioactive compounds through selective breeding and genetic modification [194]. Mushroom spawns of superior quality are crucial for successful mushroom farming [195]. Spawn production methods have improved with advances that have increased the efficiency and consistency of mushroom farming [196]. Integrated pest management (IPM) methods, such as the use of biocontrol agents and natural pesticides, minimize the use of chemical pesticides, thereby ensuring ecological sustainability [197].

### 8.3. Economic and Ecological Benefits of Sustainable Practices

The sustainable cultivation of high-ergosterol mushrooms offers numerous economic and ecological benefits, making them an ideal choice for rural development and environmental protection. Mushroom farming generates income for rural populations, particularly in areas with limited agricultural prospects [198]. High-ergosterol mushrooms can be transformed into value-added products, including dried mushrooms, extracts, and supplements, thus enhancing their market value [199]. There is an increasing international demand for medicinal and nutraceutical mushrooms, providing potential export opportunities for Himalayan mushrooms [200]. Agricultural waste can serve as a substrate for growing mushrooms, thereby minimizing waste and facilitating recycling [201]. The spent mushroom substrate can be utilized as an organic fertilizer, thereby enhancing soil health and reducing the need for chemical fertilizers [202]. Sustainable cultivation of mushrooms helps conserve indigenous species of fungi and their habitats. Farms in Himachal Pradesh have adopted sustainable farming techniques, including the utilization of crop waste as a substrate and co-cultivation with other crops. It has enhanced soil health, boosted farm returns, and lowered the environmental footprint. The sustainable production of high-ergosterol mushrooms in the Himalayas has vast economic development and ecological conservation potential. New advances in cultivation methods, including controlled environment agriculture, substrate optimization, and integrated pest management, have enhanced yield and quality. Moreover, sustainable production offers numerous economic and ecological benefits, and mushroom production is both profitable and feasible for rural communities. Additional research and investment in technology and infrastructure are necessary to fully realize the potential of this sector.

## 9. Conclusions

This review highlights the untapped potential of ergosterol-rich wild mushrooms from the Himalayas as valuable sources for nutrition and pharmacology. Ergosterol serves as a precursor to Vitamin D_2_. It exhibits antioxidant, anti-inflammatory, and immunomodulatory properties, demonstrating promise for therapeutic applications and making it important for biomedical research. However, there are significant gaps in research regarding species-level profiling, regional comparisons, and standardized growing methods. To fully utilize the benefits of ergosterol, we need interdisciplinary approaches that incorporate ethnomycology, environmental science, analytical chemistry, and biotechnology. The Himalayan region is a unique setting for this kind of research. Future efforts should focus on thorough documentation, quantifying ergosterol, and valuing local fungal diversity through sustainable development strategies that promote local biodiversity and ecosystem health. This approach can improve public health by providing natural sources of Vitamin D and help conserve Himalayan ecosystems and traditional knowledge. The Himalayan region has a diverse range of fungi and a rich cultural history. This presents a unique opportunity to enhance ergosterol research and its applications. By focusing on conservation, sustainable use, and new research, can fully tap into these natural resources for the benefit of local communities and the global population. By encouraging scientists, policymakers, and stakeholders to collaborate in preserving and utilizing Himalayan mushrooms for a healthier and more sustainable future.

## Figures and Tables

**Table 1:** State-wise compilation of the rich diversity of edible mushrooms across the Indian Himalayan Region (IHR).

Area	Division	Wild Edible Mushroom Species	Reference
Uttarakahnd	Basidiomycota	*Agaricus campestris*, *Agaricus augustus*, *Agaricus arvensis*, *Agaricus micromegathus*, *Agaricus silvaticus*, and *Agaricus silvicola*, alongside *Amanita ceciliae*, *Amanita chepangiana*, *Amanita hemibapha*, *Amanita vaginata*, *Amaria fennica* and *Astraeus hygrometricus*, *Auricularia auricula-judae* (Bull.) Quel, *Auricularia polytricha*, *Boletus edulis*, *Cantharellus cibarius Khajjiar*, *Cantharellus lateritius* (Berk.) Singer, and *Cantharellus minor*, *Chlorophyllum rachodes*, *Clavaria zollingeri*, *Clavatia craniformis*, *Coprinus comatus* (Mul.) Pers, *Craterellus cornucopioides*, *Gomphus clavatus*, *Grifola frondosa* (Dicks.) Gray, *Gymnopilus junonius*, *Hericium coralloides*, *Hericium erinaceus*, *Kuehneromyces mutabilis*, and *Lactarius* species such as *Lactarius azonites*, *Lactarius camphoratus*, *Lactarius corrugis*, *Lactarius deliciosus* (Fries) S.F. Grey, *Lactarius hygrophoroides* var. *hygrophoroides*, *Lactarius subindigo*, and *Lactarius volemus* (Fr.) Fr., Closely related, the *Lactifluus* genus features *Lactifluus corrugis*, *Lactifluus hygrophoroides*, and *Lactifluus volemus*, while the striking *Laetiporus sulphureus* (Bull.) Murrill is also included. *Macrolepiota procera*, *Macrolepiota rhachodes*, *Monotropa unifora*, *Pleurotus cornucopiae*, *Pleurotus ostreatus*, *Psathyrella candolleana*, *Ramaria botrytis* (Pers.) Ricken, *Ramaria flava*, *Ramaria sanguinea*, *Russula brevipes*, *Russula cyanoxantha* (Schaeff.) Fr., *Russula lepida*, *Russula virescens*, *Sparassis crispa* (Wulfen) Fr., *Strobilomyces floccopus*, and *Stropharia rugosoannulata*. Finally, the termite-associated *Termitomyces* species— *Termitomyces heimii* Natarajan, *Termitomyces eurrhizus* (Berk.) R. Heim, and *Termitomyces microcarpus*, *Termitomyces* sp., *Tremella foliacea*, *Tremella mesenterica*, and *Termitomyces robustus*.	[60,61,62,63,64,65]
	Ascomycota	*Aleuria aurantia* (Pers.) Fuckel, *Cordyceps sinensis*, *Helvella crispa* (Scop.) Fr., *Hydnum repandum*, *Morchella esculenta* (L.) Pers.	[60,63,64,66]
Sikkim	Basidiomycota	*Amanita vaginata*, *Auricularia auricula-judae* (Bull.) Quel, *Armillaria mellea* (Vahl) P. Kumm, *Cantharellus cibarius Khajjiar*, *Coprinus comatus* (Mul.) Pers., *Coprinus micaceus* (Bull). Fr, *Crepedotus mollis* (Schaeff. Ex. Fr.) Kumm, *Entoloma lividoalbum* (Kuhner & Romagn.) Kubicka, *Flammulina velutipes* (Curtis) Singe, *Fistulina hepatica* (Schaeff.) With., *Grifola frondosa* (Dicks.) Gray, *Hygrocybe miniata* (Fr.) Kumm., *Lactarius volemus* (Fr.) Fr, *Laetiporus sulphureus* (Bull.) Murrill, *Lentinula edodes* (Berk.) Pegler, *Lycoperdon pyreforme*, *Meripilus giganteus* (Pers.) P. Karst., *Oudemansiella mucida* (Schrad.) Hohn., *Pholiota aurivella* (Batsch) P. Kumm., *Pleurotus flabellatus* Sacc., *Ramaria subalpina*, *Ramaria aurea* (Schaeff.) Quel., *Ramaria thindii*, *Russula cyanoxantha* (Schaeff.) Fr., *Russula gnathangensis*, *Schizophyllum commune* Fr., *Sparassis crispa* (Wulfen) Fr., *Termitomyces medius* R. Heim & Grasse, *Termitomyces eurrhizus* (Berk.) R. Heim, and *Xerula radicata* (Relhan) Dorfelt.	[58,67,68]
	Ascomycota	*Aleuria aurantia* (Pers.) Fuckel	[67]
Himachal Pradesh	Basidiomycota	*Agaricus campestris*, *Agaricus comtulus*, *Agaricus fulva*, and *Agaricus silvicola*, *Amanita bisporigera* G.F. Atk., *Alloclavaria purpurea* (Fr.) Dentinger & D.J. McLaughlin, *Amanita caesarea*, *Amanita chepangiana*, *Amanita hemibapha*, *Amanita vaginata*, *Astraeus hygrometricus*, *Auricularia auricula-judae* (Bull.) Quel, *Auricularia polytricha*, *Cantharellus species*, *Cantharellus cibarius* Khajjiar, *Cantharellus lateritius* (Berk.) Singer, *Cantharellus minor*, *Conocybe tenera*, *Termitomyces microcarpus* and *Termitomyces* sp.	[66,69,70]
	Ascomycota	*Morchella deliciosa* Fries, *Morchella esculenta* (L.) Pers	[66,69,70]
Jammu and Kashmir	Basidiomycota	*Agaricus bisporus*, *Agaricus californicus* Peck, *Agaricus campestris*, *Auricularia auricula-judae* (Bull.) Quel, *Amanita vaginata*, *Bovista plumbea*, *Clavatia bovista* (L.) Pers., *Langermannia gigantea*, *Geastrum saccatum* Fr., *Calocera viscosa*, *Hericium coralloides*, *Coprinus atramentarius*, *Coprinus comatus* (Mul.) Pers., *Coprinus micaceus* (Bull.) Fr., *Flammulina velutipes* (Curtis) Sing., *Hevella lacunosa*, *Inocybe Lactarius deliciosus* (Fries) S.F., *Lentinus tigrinus*, *Leucoagaricus rhodocephalus* (Berk.) Pegler, *Lepiota procera* (Scop.) Gray, *Phallus impudicus*, *Podaxis pistillaris* (Peck) Hesler, *Ramaria formosa*, *Russula aeruginea*, *Russula aurea* Pers., *Russula cyanoxantha* (Schaeff.) Fr., *Russula delica*, *Termitomyces eurrhizus* (Berk.) R. Heim, *Termitomyces clypeatus* R. Heim, *Termitomyces heimii* Natarajan.	[71,72,73,74]
	Ascomycota	*Gyromitra esculenta*, *Gyromitra sphaerospora*, *Helvella macropus*, *Morchella esculenta* (L.) Pers., *Morchella vulgaris*, *Peziza repanda*, *Termitomyces* sp., and *Termitomyces striatus* var. *annulatus* R. Heim	[71,73]
Ladakh	Basidiomycota	*Lactarius controversus* and *Lactarius drassinus*, *Laetiporus sulphureus* (Bull.) Murrill, *Pleurotus shentelii*	[75,76,77]
	Ascomycota	*Morchella angusticipes*, *Morchella conica*, *Morchella crassipes*, *Morchella elata*, *Morchella esculenta* (L.) Pers., *Morchella deliciosa* (Fries) S.F. Grey, *Morchella gigaspora*, *Morchella hybrida*, *Morchella rotunda*, *Morchella semilibera*, and *Morchella tomentosa*	[78]
Tripura	Basidiomycota	*Lentinus tuber-regium* (Fr.) Fr., *Macrocybe gigantea* (Massee) Pegler & Loddge, *Pleurotus squarrosulus* (Mont.) Sing. *Pleurotus* genus, *Schizophyllum commune* Fr.	[59]
	Ascomycota		
Arunachal Pradesh	Basidiomycota	*Auricularia* sp., *Auricularia auricula-judae*, *Pleurotus pulmonarius*, *Polyporus squamosus*, *Pleurotus sajor-caju*, *Schizophyllum commune* Fr., *Termitomyces robustus*, *Termitomyces robustus*, *Termitomyces* sp., *Tricholoma lobayense*, *Tremella fuciformis*, and *Volvariella bombycena*	[79]
	Ascomycota	*Aleuria aurantia* (Pers.) Fuckel	[79]

## Data Availability

No new data were created or analyzed in this study. Data sharing is not applicable to this article.
